# Urinary excretion rate and bioavailability of chlorogenic acid, caffeic acid, *p*-coumaric acid, and ferulic acid in non-fasted rats maintained under physiological conditions

**DOI:** 10.1016/j.heliyon.2019.e02708

**Published:** 2019-11-01

**Authors:** Kunihiro Kishida, Harumi Matsumoto

**Affiliations:** Department of Science and Technology on Food Safety, Kindai University, Japan

**Keywords:** Biochemistry, Molecular biology, Physiology, *p*-Coumaric acid, Chlorogenic acid, Non-fasted rats, Caffeic acid, Urinary excretion, Ferulic acid

## Abstract

Hydroxycinnamic acids (HAs) are one of the major classes of phenolic compounds and epidemiological studies have suggested that they have beneficial health effects. This study aimed to determine the urinary excretion rate of chlorogenic acid, caffeic acid, *p*-coumaric acid, and ferulic acid in non-fasted rats and to estimate their bioavailability under physiological conditions. Previous studies have primarily used fasted animals, which exhibit severe changes in various physiological processes. Furthermore, the food matrix can affect HA bioavailability. Thus, our studies using non-fasted rats under physiological conditions may allow for a more accurate determination of both the HA urinary excretion rate and the bioavailability of HAs. HAs were successively gavaged to rats at a dose of 40 mg/kg body weight (BW) with a wash-out period of one week. The rats were fed the AIN-93M diet throughout the experiment. The urine was collected at time intervals of 0–6 h, 6–24 h, and 24–48 h after HA administration. Ingested HAs, except chlorogenic acid, were primarily excreted in the urine within 0–6 h as free forms or conjugated (glucuronidated and/or sulfated) forms. The majority of the ingested chlorogenic acid was detected in the urine at 6–24 h or 24–48 h as caffeic acid, *p*-coumaric acid, ferulic acid, and their conjugates. The total urinary excretion rate (% of the dose) at 48 h was ferulic acid (73.2%) > caffeic acid (61.6%) > *p*-coumaric acid (54.1%) >> chlorogenic acid (4.9%). The percentages of the conjugates in the urine differed amongst the rats gavaged with the individual HAs (74% for chlorogenic acid, 83% for caffeic acid, 68% for *p*-coumaric acid, and 96% for ferulic acid), which may be explained by their distinct bioactivities. These data reveal that caffeic acid, *p*-coumaric acid, and ferulic acid are much more bioavailable than chlorogenic acid, even though they are excreted more rapidly than chlorogenic acid. Our findings may provide additional insight into the health benefits of HAs and how they function in the body.

## Teaser

Biochemistry; Molecular biology; Physiology; Coumaric acid; Chlorogenic acid; Non-fasted rats; Caffeic acid; Urinary excretion; Ferulic acid

## 1 Introduction

Epidemiological studies have suggested that dietary phenolic compounds from plant products contribute to the prevention of degenerative diseases such as cardiovascular disease and cancer [1, 2, 3]. The antioxidant capacity of dietary phenolic compounds may explain the health benefits since several *in vitro* studies demonstrated that these compounds protect low-density lipoprotein (LDL) from oxidative modifications [4, 5, 6]. However, studies analyzing the antioxidant capacity of phenolic compounds *in vivo* are controversial, since many of the phenolic compounds are poorly absorbed and metabolized into inactive forms, resulting in low blood concentrations [7]. The United States Department of Agriculture's (USDA's) Nutrient Data Laboratory (NDL) removed the USDA ORAC database for selected foods from the NDL website because of the lack of evidence that antioxidant capacity is relevant to human health [8]. The function of phenolic compounds may extend beyond their antioxidant capacity. For example, specific phenolic compounds exhibit bioactivity *in vivo,* functioning as inhibitors of NADPH oxidase and 5-lipoxygenase [9, 10]. Phenolic compounds need to be absorbed to exert their function *in vivo* except when they act in the gastrointestinal tract. The majority of phenolic compounds in foods are present as bound forms with low bioaccessibility; thus, they are poorly bioavailable [11, 12, 13, 14]. However, various processing technologies including physical and biological treatments have been developed to improve both the bioaccessibility and bioavailability of phenolic compounds [11, 12, 13, 14]. Thus it is essential to study their bioavailability to assess their bioactive health effects.

Hydroxycinnamic acids (HAs) represented by caffeic acid, *p*-coumaric acid, and ferulic acid are one of the major classes of phenolic compounds that are abundant in fruits, vegetables, cereals, and coffee [11, 12, 13, 14, 15, 16]. Many studies have been conducted to measure the bioavailability of HAs using pharmacokinetic profiles and urinary excretion in animal models and humans [17, 18]. However, the experimental designs were significantly different among the studies. A few reports focused on one type of HA and other studies determined only free forms of HAs in plasma or urine [17, 18]. Additionally, rats were often fasted for 12–24 h before administration of HAs to minimize individual differences [19, 20, 21, 22]. Fasting induces severe changes in the physiological and biochemical processes of the animal, which become more serious with longer duration [23]. In addition, Adam et al. showed that the bioavailability of ferulic acid is governed primarily by the food matrix [24]. Thus, it is difficult to compare and review the results obtained from different studies that do not share the same experimental conditions. Therefore, the aim of this study was to determine the urinary excretion rate of representative HAs, including chlorogenic acid, caffeic acid, *p*-coumaric acid, and ferulic acid in non-fasted rats and to estimate their bioavailability under physiological conditions.

## 2 Materials and methods

### 2.1 Chemicals

The following analytical grade chemicals were used in this study: acetic acid, ascorbic acid, propylene glycol, sodium acetate, and trifluoroacetic acid. HPLC grade methanol was also utilized in this study. The aforementioned chemicals were purchased from Wako Pure Chemical Industries, Ltd. (Osaka, Japan). The following analytical grade chemicals used in this study were purchased from Sigma-Aldrich Co. LLC (St. Louis, MO, USA): caffeic acid, chlorogenic acid, *p*-coumaric acid, ferulic acid, and sinapinic acid. β-GFlucuronidase (EC 3.2.1.3 1) type H-2 and type X-A were also purchased from Sigma-Aldrich Co. LLC (St. Louis, MO, USA). Water was purified using a Milli-Q system (Millipore, Bedford, MA, USA). The DISMIC-13_HP_ 0.20 μm (hydrophilic PTFE) disposable syringe filter units were purchased from Toyo Roshi Kaisha, Ltd. (Tokyo, Japan).

### 2.2 Animals, diets and experimental design

Male Wistar rats (n = 4, 21–25 weeks old, Kiwa Laboratory Animals Co. Ltd., Wakayama, Japan) were housed singly in metabolic cages in a temperature-controlled room (21 °C) under a 12 h light-12 h dark cycle and were fed the AIN-93M diet (Oriental Yeast Co., Ltd., Tokyo, Japan). Without fasting, 40 mg/kg BW of caffeic acid, *p*-coumaric acid, ferulic acid, or chlorogenic acid dissolved in 10% propylene glycol was successively gavaged to the rats with a wash-out period of one week. Urine was collected in bottles containing 100 μL of 100 mg/mL ascorbic acid and sodium azide solution at time intervals of 0–6 h, 6–24 h, and 24–48 h after administration of the HA. The urine samples were stored at -80 °C until analysis. The rats were fed the AIN-93M diet throughout the experiment. This study was approved by the Animal Care Committee of Kindai University (permit number KABT-24-002), and the animals were maintained in accordance with the guidelines. Sample size, dose, and duration of urine collection were determined on the basis of our preliminary experiments.

### 2.3 Treatment of urine samples and HPLC analyses

The HA composition in urine was determined by the method described by Zhao et al. with a modification [25]. In our preliminary experiment, the modified procedure was validated by a recovery test (data not shown). The urine sample (20 μL) was added to 360 μL of acetic acid buffer (pH 5.0) containing 10 μg/mL sinapinic acid (internal standard) and 20 μL of acetic acid buffer (pH 5.0). The mixed solution was filtered through a 0.20 μm disposable syringe filter unit. The filtrate was analyzed by HPLC to quantify free forms of the HAs. Enzymatic hydrolysis was used to determine conjugated forms (glucuronidated and/or sulfated forms). The urine sample (20 μL) was added to 360 μL of acetic acid buffer (pH 5.0) containing 10 μg/mL sinapinic acid (internal standard) and 20 μL of β-glucuronidase type H-2 (containing both β-glucuronidase and sulfatase activity). For determination of chlorogenic acid levels, β-glucuronidase type X-A was used instead of type H-2 because type H-2 has chlorogenic acid esterase activity. The mixture was saturated with nitrogen gas and incubated at 37 °C for 2 h, followed by filtration through a 0.20 μm disposable syringe filter unit. The filtrate was analyzed by HPLC and the total amount of HAs (free forms and conjugated forms) was quantified. The conjugated HA levels were calculated by subtracting the amount of HA free forms from the total amount of HAs. The HPLC system comprised a LC-2010 equipped with an SPD-M10Avp photodiode array detector (Shimadzu Corporation, Kyoto, Japan) with a Hydrosphere C18 column (φ 4.6 mm × 250 mm; YMC CO., LTD, Kyoto, Japan). The separation was performed using a 0.1% (v/v) trifluoroacetic acid–methanol solution (75:25, v/v) as the mobile phase at a flow rate of 1.0 mL/min at 40 °C. The injection volume was 10 μL. *p*-Coumaric acid was detected at 309 nm and the other HAs as well as sinapinic acid were detected at 325 nm based on the maximum absorbance. The urinary excretion rate was calculated as the percentage of the dose (mol/mol).

### 2.4 Statistical analyses

All data were expressed as the mean ± SE for each group. The statistical analysis was performed with an unpaired Student's t-test or a one-way ANOVA with Tukey's multiple range test using the IBM SPSS statistics software, version 19.0 (IBM Co., New York, NY, USA). P-values < 0.05 were considered statistically significant and are indicated by a single asterisk (*) or different letters.

## 3 Results

**Fig. 1:**
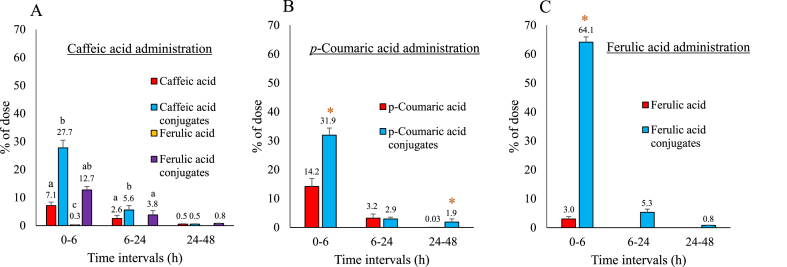
The urinary excretion rate (% of the dose) of caffeic acid (A), *p*-coumaric acid (B), and ferulic acid (C) after oral administration to non-fasted rats. The data represent the mean ± SE (n = 4). Forty mg/kg BW of caffeic acid, *p*-coumaric acid, or ferulic acid was gavaged to the non-fasted rats. The rats were fed the AIN-93M diet throughout the experiment. The urine was collected at time intervals of 0–6 h, 6–24 h, and 24–48 h after HA administration. The numbers above the bars comprise the mean values. A single asterisk (*) and different letters indicate statistically significant differences (P < 0.05).

Fig. 1 shows the HA urinary excretion rates in non-fasted rats at time intervals of 0–6 h, 6–24 h, and 24–48 h after gavage with caffeic acid, *p*-coumaric acid, or ferulic acid. When caffeic acid was gavaged to the rats, intact caffeic acid (7.1% of the dose), ferulic acid (0.3% of the dose), and significantly higher amounts of caffeic acid conjugates (27.7% of the dose) as well as ferulic acid conjugates (12.7% of the dose) were found in the urine at 0–6 h (Fig. 1A). The total urinary excretion of caffeic acid at 48 h was 61.6 ± 2.0% of the dose, of which 83% was conjugates. The majority of the ingested caffeic acid was excreted at 0–6 h (Fig. 1A, Table 1).

**Table 1:** Total urinary excretion rate (% of the dose) of chlorogenic acid, caffeic acid, *p*-coumaric acid, and ferulic acid at 48 h after oral administration in non-fasted rats.

	Compounds excreted in the urine at 48 h (% of the dose)								Total
	Chlorogenic acid	Chlorogenic acid conjugates	Caffeic acid	Caffeic acid conjugates	*p*-Coumaric acid	*p*-Coumaric acid conjugates	Ferulic acid	Ferulic acid conjugates	
Compounds orally administrated									
Chlorogenic acid	0.04 ± 0.005^a^	0.05 ± 0.006^a^	0.73 ± 0.03^b^	0.22 ± 0.02^ab^	0.50 ± 0.04^ab^	1.47 ± 0.15^c^	N.D.	1.88 ± 0.13^c^	**4.9 ± 1.0**
Caffeic acid	N.D.	N.D.	10.2 ± 0.82^a^	33.8 ± 2.94^b^	N.D.	N.D.	0.26 ± 0.03^c^	17.3 ± 1.35^d^	**61.6 ± 2.0**
*p*-Coumaric acid	N.D.	N.D.	N.D.	N.D.	17.4 ± 2.0^a^	36.7 ± 4.1^b^	N.D.	N.D.	**54.1 ± 5.2**
Ferulic acid	N.D.	N.D.	N.D.	N.D.	N.D.	N.D.	3.01 ± 0.38^a^	70.2 ± 6.8^b^	**73.2 ± 2.1**

**Fig. 2:**
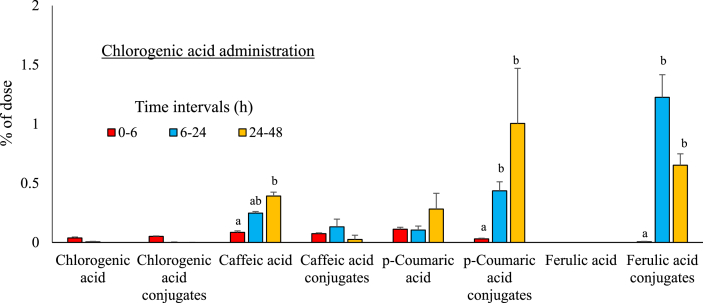
The urinary excretion rate (% of the dose) of chlorogenic acid after oral administration to non-fasted rats. The data represent the mean ± SE (n = 4). Forty mg/kg BW of chlorogenic acid was gavaged to the non-fasted rats. The rats were fed the AIN-93M diet throughout the experiment. The urine was collected at time intervals of 0–6 h, 6–24 h, and 24–48 h after chlorogenic acid administration. The total excretion amount was 0.4% at 0–6 h, 2.2% at 6–24 h, and 2.4% at 24–48 h. The different letters indicate statistically significant differences (P < 0.05).

Data are represented as the mean ± SE (n = 4). See Fig. 1 and Fig. 2 for the time profile.

Different letters indicate statistically significant differences (P < 0.05).

N.D., not detected.

When *p*-coumaric acid was gavaged to the rats, intact *p*-coumaric acid (14.2% of the dose) and significantly higher amounts of its conjugates (31.9% of the dose) were found in the urine at 0–6 h (Fig. 1B). *p*-Coumaric acid exhibited maximum excretion at 0–6 h. The total urinary excretion of *p*-coumaric acid was 54.1 ± 5.2% of the dose at 48 h (Fig. 1B, Table 1). The *p*-coumaric acid conjugates accounted for 68% of the total excreted *p*-coumaric acid.

Intact ferulic acid (3.0% of the dose) and significantly higher amounts of its conjugates (64.1% of the dose) were found in the urine at 0–6 h in the rats gavaged with ferulic acid (Fig. 1C). The total urinary excretion of ferulic acid was 73.2 ± 2.1% of the dose at 48 h, most of which (96%) was conjugated and excreted at 0–6 h (Fig. 1C, Table 1).

In contrast to the above 3 types of HAs, rats gavaged with chlorogenic acid exhibited much lower urinary excretion. Chlorogenic acid, caffeic acid, *p*-coumaric acid, ferulic acid, and their conjugated forms were detected in the urine (Fig. 2, Table 1). Intact chlorogenic acid (0.04% of the dose) and its conjugates (0.05% of the dose) were found in the urine at 48 h. Intact caffeic acid, *p*-coumaric acid, ferulic acid, and their conjugates were excreted in the range of 0–1.2%, with the majority found in the urine at 6–24 h and 24–48 h after administration, which is much later compared to other HAs. The conjugates accounted for 74% of the total excretion.

## 4 Discussion

The aim of this study was to determine the urinary excretion rates of representative HAs in non-fasted rats under the same conditions and to estimate their bioavailability under physiological conditions. In the present study, the urinary excretion rate was ferulic acid > caffeic acid > *p*-coumaric acid >> chlorogenic acid as summarized in Table 1. The results suggest that the majority of ingested caffeic acid, *p*-coumaric acid, and ferulic acid were absorbed, conjugated, and excreted in the urine within 0–6 h, while chlorogenic acid was poorly absorbed and urinary excretion was detected at 6–24 h and 24–48 h. Konishi et al. reported that the intestinal absorption of these HAs was mediated by monocarboxylic transporters (MCTs) and plasma HA concentrations were associated with the order of affinity for MCTs (ferulic acid = *p*-coumaric acid > caffeic acid > chlorogenic acid) [26, 27, 28, 29] Our results are in agreement with these studies except that in our studies caffeic acid exhibited a higher urinary excretion rate than *p*-coumaric acid. Thus, the HAs examined in this study were likely absorbed via MCTs and the different affinity for MCTs likely resulted in the different bioavailabilities. The discrepancy in the caffeic acid results between the present study and previous reports may be due to different experimental approaches. Here, we determined the urinary excretion rates in non-fasted rats, whereas other studies analyzed plasma HA concentrations in pylorus-ligated rats or measured HA permeability using Caco-2 cells [26, 27, 28, 29]. In addition, we found ferulic acid conjugates equivalent to 17.3% of administered caffeic acid, although Konishi et al. did not determine the ferulic acid conjugate levels in plasma [26]. Our results suggest that ingested caffeic acid was rapidly absorbed and mainly conjugated and/or methylated into ferulic acid, followed by excretion in the urine. Gonthier et al. reported that rats fed a diet supplemented with caffeic acid exhibited a total urinary excretion rate of 12.8% of intake. They also found that the excreted HAs consisted of caffeic, ferulic, and isoferulic acids as well as their conjugates [30]. Garrait et al. reported that 23% of ingested *p*-coumaric acid was found in the urine as an intact form using fasted rats, and the majority of *p*-coumaric acid was detected within 4 h after gavage, which is generally consistent with our results [20]. In addition, similar to our results, others have reported that 50–70% of ingested ferulic acid was recovered as conjugated forms in the urine [24, 25]. It is interesting that fasting and non-fasting conditions showed a similar urinary excretion pattern.

We observed much lower and slower urinary excretion of chlorogenic acid compared to other HAs (Figs. 1 and 2). In addition, no fecal HAs were detected in our preliminary experiments (data not shown). These findings support the following conclusions: (1) a small portion of chlorogenic acid was absorbed into the small intestine in its intact form and the remainder entered the cecum and large intestine; (2) caffeic acid, derived from the hydrolysis of chlorogenic acid by microflora, was methylated into ferulic acid in tissue; and (3) *p*-coumaric acid was formed by microbial dehydroxylation of caffeic acid as previously reported [19, 31, 32, 33, 34, 35, 36, 37]. Interestingly, comparing the results obtained from chlorogenic acid and caffeic acid administration, *p*-coumaric acid was detected only after chlorogenic acid administration, suggesting that *p*-coumaric acid was formed by a quinic acid moiety of chlorogenic acid by microflora. Gonthier et al. reported that the urinary excretion of chlorogenic acid or the total amount of caffeic, ferulic, and isoferulic acids was less than 1% of the chlorogenic acid intake, which is similar to our results [30]. However, they detected *p*-coumaric acid and *m*-coumaric acid in the urine of rats fed diets supplemented not only with chlorogenic acid but also with caffeic acid, although a small amount of *p*-coumaric acid was also found in the rats fed diets supplemented with quinic acid. *p*-Coumaric acid and *m*-coumaric acid are microbial metabolites, while ferulic acid is a tissue metabolite [33, 34, 37]. Therefore, the discrepancy could be explained by differences in the microflora population. Further investigation is required to identify the microorganisms that can produce these metabolites.

When considering the bioactive health effects of HAs, it should be noted that the percentages of conjugates in the urine differed amongst the rats gavaged with the HAs (74% for chlorogenic acid, 83% for caffeic acid, 68% for *p*-coumaric acid, and 96% for ferulic acid, Table 1). This may be explained by the different biological properties of the conjugates. In general, the conjugates have less bioactivity than the intact forms; however, a few of them have greater bioactivity [10, 38, 39]. Further research is required to evaluate the health effects of the HA conjugates. This study evaluated the urinary excretion profile of HAs (chlorogenic acid, caffeic acid, *p*-coumaric acid, and ferulic acid) under the same conditions and measured their bioavailability under physiological conditions. Our results showed that caffeic acid, *p*-coumaric acid, and ferulic acid are much more bioavailable than chlorogenic acid, even though they are excreted more rapidly than chlorogenic acid. These findings may advance our understanding of the beneficial health effects of bioactive HAs that act in the body.

## Declarations

### Author contribution statement

K. Kishida: Conceived and designed the experiments; Performed the experiments; Analyzed and interpreted the data; Contributed reagents, materials, analysis tools or data; Wrote the paper.

H. Matsumoto: Performed the experiments; Analyzed and interpreted the data; Contributed reagents, materials, analysis tools or data.

### Funding statement

This research did not receive any specific grant from funding agencies in the public, commercial, or not-for-profit sectors.

### Competing interest statement

The authors declare no conflict of interest.

### Additional information

No additional information is available for this paper.
